# Mechanical time-of-flight filter based on slotted disks and helical rotor for measurement of velocities of nanoparticles

**DOI:** 10.1038/s41598-021-85533-7

**Published:** 2021-03-19

**Authors:** Pavel Solař, Jaroslav Kousal, Jan Hanuš, Kateřina Škorvánková, Anna Kuzminova, Ondřej Kylián

**Affiliations:** Department of Macromolecular Physics, Faculty of Mathematics and Physics, Charles University, Prague, 182 00 Czech Republic

**Keywords:** Materials science, Nanoscience and technology, Physics

## Abstract

A mechanical time-of-flight filter intended for measurement of velocities of nanoparticles exiting a gas aggregation source has been developed. Several configurations maximizing simplicity, throughput or resolution are suggested and investigated both theoretically and experimentally. It is shown that the data measured using such filters may be easily converted to the real velocity distribution with high precision. Furthermore, it is shown that properly designed filters allow for the monitoring of the velocity of nanoparticles even at the conditions with extremely low intensity of the nanoparticle beam.

## Introduction

Gas-phase synthesis of nanoparticles (NPs) by magnetron-based Gas Aggregation Sources (mGAS) has established itself as one of the most viable strategies of preparation of this class of nanomaterials in the last few decades^1–3^. The popularity of this vacuum-based and fully solvent-free deposition technique developed in the 1990s^4,5^, which is based on the spontaneous nucleation of sputtered material accompanied by subsequent coalescence and coagulation of the growing particles, is primarily due to the possibility of fabricating high-purity nanoparticles with well-controlled size and morphology. As shown in numerous studies, mGAS systems are also very flexible concerning the materials from which the nanoparticles can be prepared that range from single-material metallic^6–17^, metal-oxide^18–20^ or plasma polymer NPs^21–26^ to multi-material ones^27–37^. Furthermore, the gas aggregation sources allow for the deposition of nanoparticles on virtually any substrate that can withstand the vacuum conditions. However, the way how the gas-phase synthesized NPs interact with a substrate and assemble on it is strongly dependent on many factors. Among them, one of the most important ones was found to be the velocity/energy of NPs upon their impact on a solid substrate. Related to this, two principal deposition regimes may be distinguished depending on the energy of incoming NPs^38,39^ that is commonly expressed as the kinetic energy per atom *E*_*at*_. While the integrity and structure of both nanoparticle and substrate may dramatically change for *E*_*at*_ higher than the binding energy of the nanoparticle constituents, nanoparticle stays intact and preserves its chemical composition during its interaction with a substrate at lower kinetic energies^40^. In addition, it has been demonstrated that in the latter case, i.e. in the so-called “soft-landing” regime, the nanoparticles may be in some cases rebound from the substrate. This process is very sensitive not only to the physicochemical properties of both the substrate and incoming nanoparticle (e.g. structure, morphology and mechanical properties of substrate and NPs) but also to relatively small variations in the velocity of nanoparticles interacting with the substrate material^41,42^. For instance, as shown in^41^ for nanoparticles with a mean diameter between 10 and 100 nm, the bouncing may occur at velocities around several tens of m/s, i.e. in the velocity range common for magnetron-based gas aggregation sources with no additional nanoparticle acceleration. The reflection of nanoparticles and its proper control in turn allowed for a highly selective nanoparticle assembly on substrates, i.e. an important step towards the production of functional nanodevices^42,43^.

Another field in which the velocity of nanoparticles has to be considered is their in-flight coating/modification. This technique utilizes the spatiotemporal separation of the core formation that takes place inside the aggregation chamber of mGAS and its subsequent modification/coating that occurs in an auxiliary zone located in between the output of mGAS and substrate. Such an approach was recently employed for the production of various types of core/shell^44,45^ and core/satellite^46,47^ nanoparticles or for the in-flight oxidation of metallic NPs^20,48^. Naturally, the critical parameter that determines the performance of this deposition strategy is the residence time of NPs in the zone, where they are modified. This is naturally given by their velocity and the length of the modification zone.

Both aforementioned examples highlight the necessity of the precise knowledge of nanoparticle velocity for the rational and effective design of the deposition procedure. Nevertheless, in contrast to carrier gas, whose speed may be easily calculated from the known geometry of the system and gas flow, the determination of the velocity of nanoparticles remains rather challenging using theoretical models as the size of the velocity slip effect is still generally unknown. Because of this, the precision of the calculations is, in many cases, questionable, and thus the direct measurement of the particle velocity is urgently needed.

The first attempt to measure the nanoparticle velocity was presented in^7,49^ using a system based on an electrostatic deflection. However, this approach has intrinsic limitations as it requires the knowledge of the mass and charge of nanoparticles, does not allow in-situ monitoring of nanoparticle velocity and is applicable solely for the charged nanoparticles. Another method, a time-gated quadrupole mass filtering^50,51^ allows in-situ monitoring, but again only of the charged NPs. In addition, such systems allow filtering of nanoparticles only in a rather narrow range of their size. The velocity of charged NPs may also be in principle measured using a Wien filter^52^ or electrostatic time-of-flight filter^53^. However, the exclusion of neutral and opposite charged NPs is too severe limitation because aside from not being able to detect all nanoparticles, the charging of NPs is size-dependent and may distort the results and complicate the interpretation of the results. A theoretical possibility to measure the velocity of NPs including neutral ones would be based on the measurement of beam impuls^54^. Aside from other technical issues connected with this strategy, the sensitivity of such measuring systems is still not sufficient even for high-intensity beam mGASes. To overcome these principal drawbacks, a novel approach is investigated in this study that utilizes a mechanical slotted disk type filter originally developed for the measurement of the velocity of molecular beams (e.g.^55–58^) and used previously as a filter for mass selection of gas-phase synthesized NPs^59^. To our best knowledge, it has never been used for the measurement of velocities of nanoparticles, and thus the main aim of this study is (i) to test the applicability of such velocity filter for the evaluation of velocities and velocity distribution function of nanoparticles generated by mGAS and (ii) to show, how the selectivity and high throughput may be optimized by a proper design of the filter.

## Experimental

### Deposition setup

**Figure 1:**
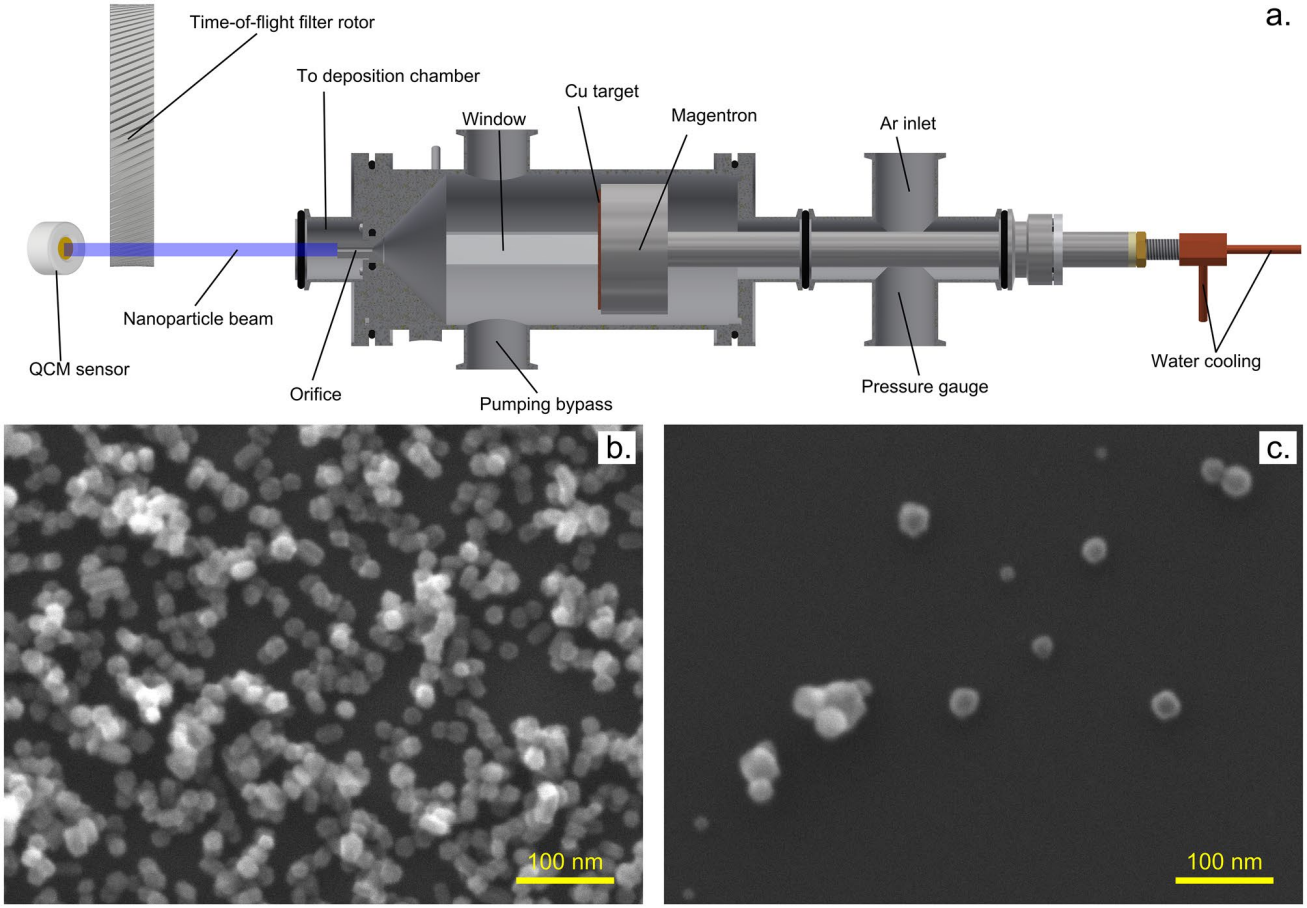
(**a**) Schematics of the nanoparticle source; (**b**) copper NPs deposited at pressure 20 Pa (mean size 18 nm, the standard deviation of mean 2.5 nm); (**c**) copper NPs deposited at pressure 100 Pa (mean size 26 nm, the standard deviation of mean 7 nm); The images have been obtained using a scanning electron microscope (SEM, JSM-7200F, JEOL). The samples have been deposited on single side polished silicon wafers and observed in secondary electron mode. The sizes have been determined by fitting NPs circumference by a circle.

To test the performance of constructed filters, a gas aggregation source shown schematically in Fig. 1a has been used. The source is similar to the one employed in our previous studies^60^. It was equipped with a magnetron 81 mm in diameter set with copper target 3 mm thick. The orifice was 23.5 mm long, 3 mm in diameter. The aggregation length, i.e. distance from the target surface to the end of the orifice, was 175 mm. The source was connected to a high vacuum main chamber pumped by diffusion and rotary pumps. The working gas was argon (99.996% purity, Linde Gas), which was introduced via a flow controller (MF-1, range 20 sccm, MKS). A capacitance gauge was used to measure pressure both in the aggregation chamber (Baratron 626C, range 133 Pa, MKS) and in the main deposition chamber (Baratron 626C, range 1330 Pa, MKS). The magnetron was powered with a DC magnetron source (MDX1.5K, Advanced Energy).

To investigate the filter performance, the NPs have been deposited at magnetron current 200 mA at two different aggregation chamber pressures 20 and 100 Pa corresponding to flowrates of the buffer gas 1 and 12.7 sccm. Under these conditions, Cu nanoparticles with mean diameters of 18 ± 3 nm and 26 ± 7 nm were produced as witnessed by scanning electron microscopy using JEOL (JSM-7200F) electron microscope operated in the secondary electron mode and 15 kV acceleration voltage (see Fig. 1b,c). Furthermore, the part of the beam of NPs leaving the mGAS that is collected on the deposition rate sensor exhibited the divergence from parallelism better than 2°.

### Velocity filter and nanoparticles detection

**Figure 2:**
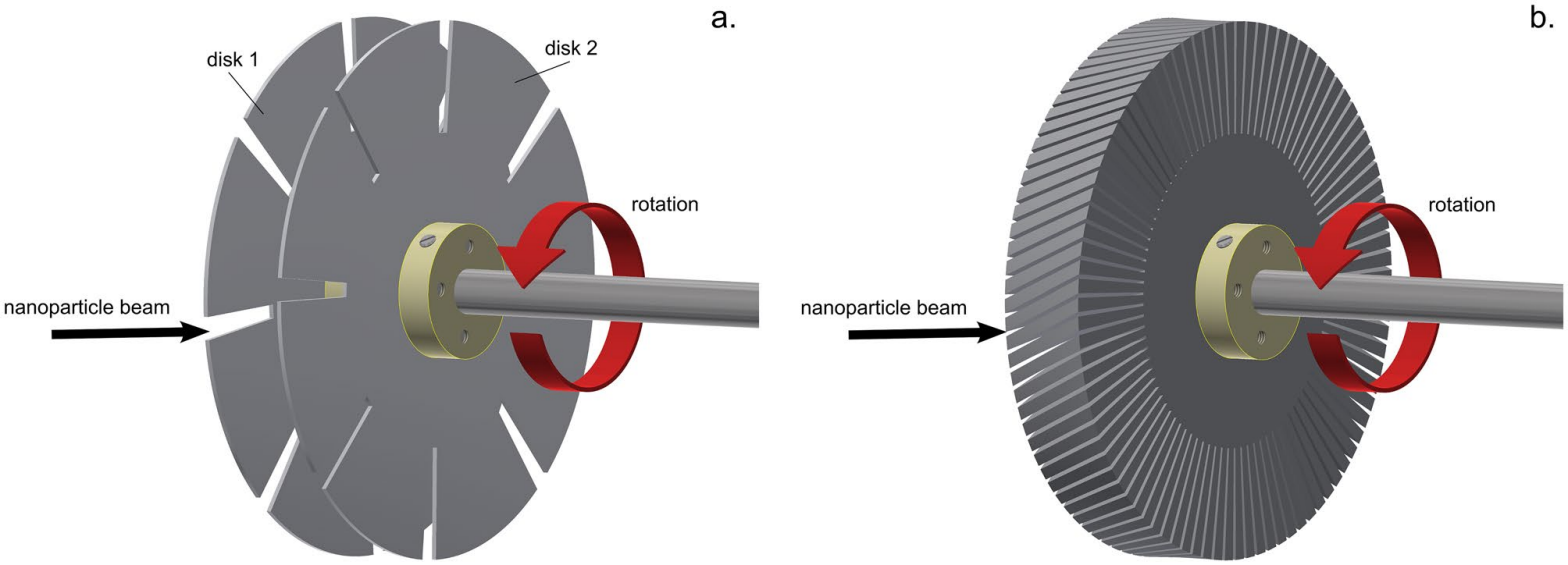
Schematics of the nanoparticle time-of-flight filter rotors: (**a**) slotted disks with 8 slits of angular width 5° on each disk. The relative angle between the disks is 9°, disks-to-disk distance is 19 mm, disk thickness is 1 mm; (**b**) helical rotor with 90 slits of angular width 1°, the relative angle between entrance and exit 9°, total thickness 20 mm and printing resolution 0.2 mm.

The basic configuration of the velocity filter, which is depicted in Fig. 2a, is based on two slotted disks 120 mm in diameter, 1 mm thick, on a common shaft connected directly to a brushless DC motor (BLDC) commonly used e.g. in turbo-molecular pumps or drones. The used motor is capable of driving the system in a frequency range from 8 to about 270 Hz. The rotation frequency was measured using an optical element. The system has 3 adjustable geometry parameters that influence the transmissivity (resolution) and measuring range. The first of these parameters is the angular width of the slots in the disks *α*; the wider is the slit, the higher is the particle transmissivity, but at the cost of lower resolution. In the experiments reported in this study, three configurations with 5, 2 and 1° wide slits have been investigated. The number of slits was kept constant at number 8, which assured that for the rotation frequency range the particles may only be transmitted through the first slit on the second disk and never any higher. The second parameter that plays an essential role in the performance of the velocity filter is the mutual distance of the disks *l*; higher distance shifts the range to higher nanoparticle velocities. For the experiments summarized in this paper, the disk-to-disk distance was fixed at 19 mm (between mid-planes of each disk). The thickness of the disks was 1 mm each (the total rotor thickness was therefore 20 mm). The third key parameter is the relative angle between the slits of the first and second disks *φ*. This should be higher than the angular width of the slit to avoid nanoparticle transmission at zero rotation. In addition, the higher the value of *φ*, the lower velocities are transmitted. In our experiments, the *φ* was fixed at 9°. The described setup with the specified geometry parameters allows measurement of nanoparticle velocity from 7 to 215 m/s.

The second and more complex variant of the rotor was based on series of layers of very thin disks set one on the top of another, each rotated by an incremental angle relative to the previous (see Fig. 2b). In this study, the rotor, which is denoted as a helical rotor in the subsequent text, had slit angular width 1° and wall angular width 3°.

Except for the central shaft, motor connector, the disk holders, ball bearings and screws, all the remaining parts of both slotted disk and helical rotor devices were 3D printed on Prusa i3 MK3S printer from PETG polymer using a 0.4 mm nozzle. The printing resolution (layer thickness) was 0.2 mm.

The transmitted nanoparticles were collected on a Quartz Crystal Microbalance sensor (QCM, own design) using 0.5-in. crystals and Ø12 mm sensor opening. The QCM frequency (5 MHz) was read using a universal frequency counter (TF930, Aim-TTi instruments) with 3 GHz reference, 1 mHz resolution and 0.1 Hz resolution repeatability, gate time 1 s and refresh rate 500 ms. All the data were logged in the computer and synchronized to other quantities, such as the actual rotation frequency and other operating conditions. To suppress noise in the case of low deposition rate measurements, the deposition rates were optionally smoothed over 5 or 20 measurements corresponding to 2.5 and 10 s measurement time. The smoothing allows noiseless detection of very low deposition rates, but the rate of rotation frequency change must be slower than the averaging time to avoid shifting or smudging of the logged data.

### Model of filter operation

**Figure 3:**
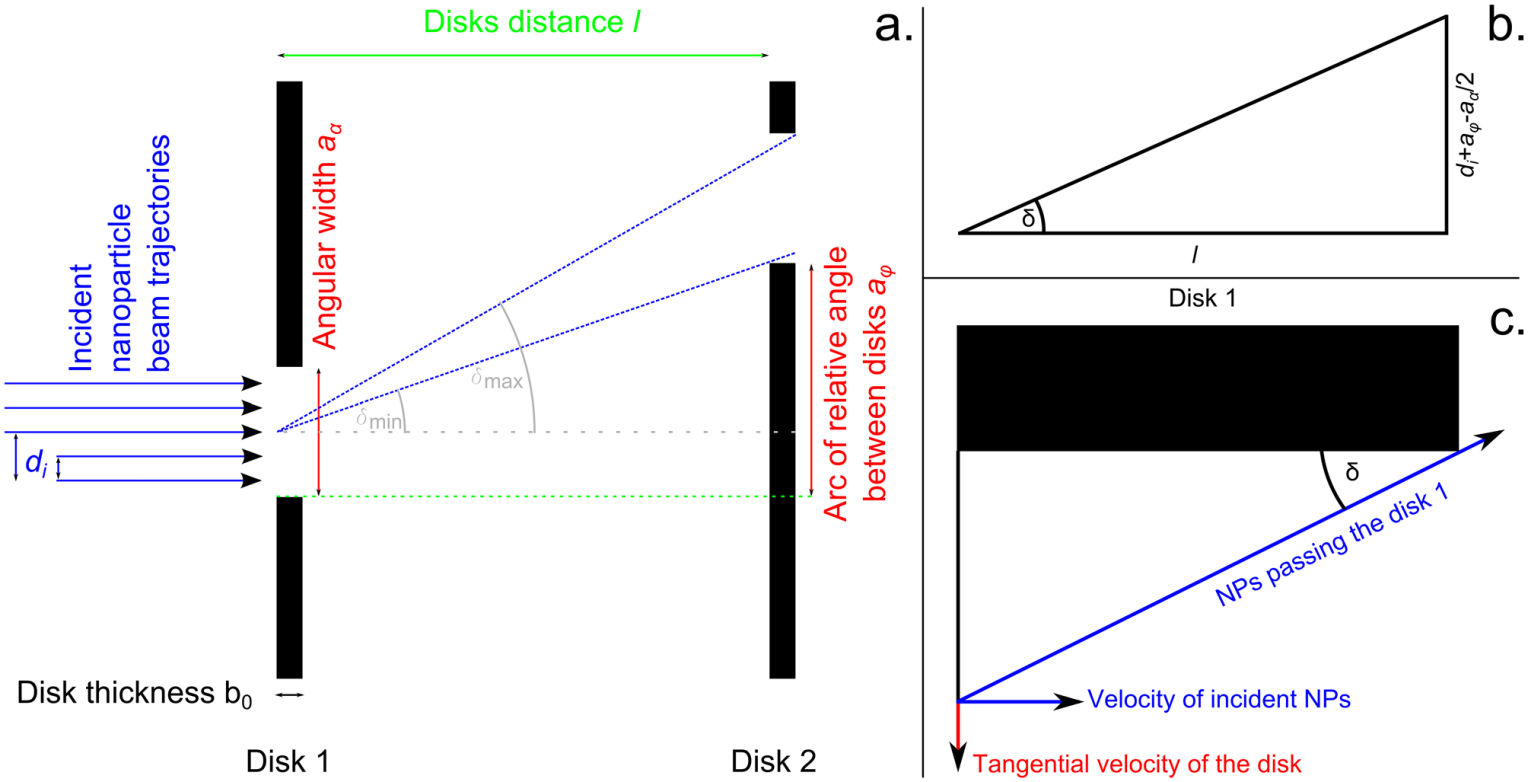
Schematic illustration of the model: (**a**) schematic of the whole system; (**b**) geometric quantities for calculation of *δ*_*max*_ or *v*_*min*_; (**c**) geometric illustration of NPs collected on the inner wall of the entrance slit/transmitted NPs in the zoomed area around the upper part of the slit in disk 1.

A semi-analytical model of the device suitable for the numerical calculation has been developed in order to fit the measured data as well as to predict the behavior of the measurement system. The basic idea of the model is to use a number of test particles travelling parallel to each other along normal to the disks planes across the entrance slit of the rotor (Fig. 3a). Each of such particles entering the velocity filter is then tested if it passes the system or not at a specific velocity and rotation rate. The particles that pass for each value of rotation rate are then summed into the signal value. The input trajectories are equidistant. Since the system is rotating, it does not matter what kind of profile the beam initially has; it becomes homogeneous across the slit.

For the purpose of the model, let’s look at the device from the surface of the cylinder, the radius of which is the distance between the rotor axis and the NPs beam, being unfold into a plane, see Fig. 3a. For the angular width of the slits *α,* the relative angle of the disks *φ* and the radius of the slit (the radius where the beam is passing) *r*_*0*_, it is possible to define the length of the arc of the slit *a*_*α*_

1 $${a}_{\alpha }=2\pi {r}_{0}\frac{\alpha }{360}$$

and the length of the arc given by relative angle of the disks *a*_*φ*_

2 $${a}_{\varphi }=2\pi {r}_{0}\frac{\varphi }{360}$$

The tangential velocity of the disks is given by

3 $${v}_{rot}=2\pi {r}_{0}f$$

where *f* represents the frequency of the disks rotation. The incident trajectory (distance from one edge of the entrance slit along the slit arc) is defined as

4 $${d_i} = \frac{{{a_\alpha }}}{2}\frac{{i - \frac{n}{2}}}{{\frac{n}{2}}}$$

where *n* + *1* is the number of input trajectories and *i* is the index of selected trajectory going from 0 to *n*.

The limits of the incident velocity of the transmitted NPs for given trajectory *d*_*i*_ and rotation frequency *f* are given by the two dashed blue lines in Fig. 3a, the angle of which relative to rotor axis (along *l*) is labelled *δ*, see Fig. 3b. The maximum velocity *v*_*max*_ of the nanoparticle to pass through the system is then given by the lower line, while the minimum velocity *v*_*min*_ by the upper line. The angle *δ* may be expressed either in terms of velocity as a ratio between tangential velocity *v*_*rot*_ of the slits and the incident NPs velocity *v*_*in*_ as

5 $$\tan \delta = \frac{{{v_{rot}}}}{{{v_{in}}}}$$

or in terms of the geometry of the system as

6 $${\tan}{\delta }_{min}=\frac{{d}_{i}+{a}_{\varphi }-\frac{{a}_{\alpha }}{2}}{l}$$

7 $${\tan}{\delta }_{max}=\frac{{d}_{i}+{a}_{\varphi }+\frac{{a}_{\alpha }}{2}}{l+{b}_{0}}$$

where *b*_*o*_ is the thickness of the disks and *l* is the distance of the centers of the disks. Note that for *δ*_*max*_ the limit beam passes the outer corner of the disk 2, while for *δ*_*min*_ it passes the inner corner. The velocity of the transmitted NPs maybe then expressed as

8 $${v}_{min}=\frac{{v}_{rot}}{{\tan}{\delta }_{max}}$$

9 $${v}_{max}=\frac{{v}_{rot}}{{\tan}{\delta }_{min}}$$

After substitution of all variables into Eqs. (8) and (9) and adjustments, the velocity limits may be rewritten as

10 $${v}_{min}=\frac{360f\left(l+{b}_{0}\right)}{\frac{\alpha }{n-1}\left(i-\frac{n-1}{2}\right)+\varphi +\frac{\alpha }{2}}$$

11 $${v}_{max}=\frac{360fl}{\frac{\alpha }{n-1}\left(i-\frac{n-1}{2}\right)+\varphi -\frac{\alpha }{2}}$$

The Eqs. (10) and (11) presented above do not consider the NPs that do not pass the first slit because they get caught by the inner wall of the entrance disk, see Fig. 3c. Although these NPs satisfy the Eqs. (10) and (11) they have to be subtracted from the result. The critical distance *d*_*crit*_ of the incident NPs trajectory from the upper slit border that allows NPs to pass may be expressed as

12 $${d}_{crit}={b}_{0}{\tan}\delta =2\pi {r}_{0}\frac{\alpha }{360}\frac{i}{n-1}$$

After substitution and adjustments the first transmitted trajectory is

13 $${i}_{crit}=\frac{360n{b}_{0}f}{\alpha {v}_{in}}$$

With Eqs. (10), (11) and (13), it is possible for specified rotation frequency *f*, NPs velocity *v* and trajectory *i* to determine whether the nanoparticle will pass or not through the system. The signal *s* at the given rotation frequency is then obtained by the sum of the blocked/passed counts *c* = {0,1} over the trajectory index and the possible NPs velocities multiplied by the probability of the specific NPs velocity *p*_*v*_.

14 $$s\left(f\right)=\sum_{v}\left({p}_{v}\sum_{i=0}^{n}{c}_{iv}\right)$$

The mean velocity transmitted through the filter assuming the finite thickness of the rotor disks may be assumed in the form

15 $${v}_{mean}=\frac{360fl}{\varphi }$$

To obtain the simulated signal, the above-described calculations have to be executed for a frequency range and the peak amplitude has to be adjusted to the measured data. A program to simulate the behavior of the system at various configurations and to fit measured data has been written in Delphi XE2.

It should be noted that other models have been developed for very similar systems and also give very similar results even though the method of derivation differs. Our model has been derived with stress on simplicity, accuracy and also to be simulated using a computer. The measurement is, in principle, a dependence of the deposition rate (signal) on the rotation frequency *f,* which is also the output of the model. By shifting from frequency to velocity and sending the number of trajectories *n* to infinity, the relation (14) gives the same result as the equation derived in^55^ under number (8). In our case, however, the assumption of constant function for NPs velocity distribution is unusable. The measurement is also principally discrete with generally unknown velocity distribution, in which case the numerical integration is the only practical choice.

## Results and discussion

### Theoretical analysis of the performance of time-of-flight filter

**Figure 4:**
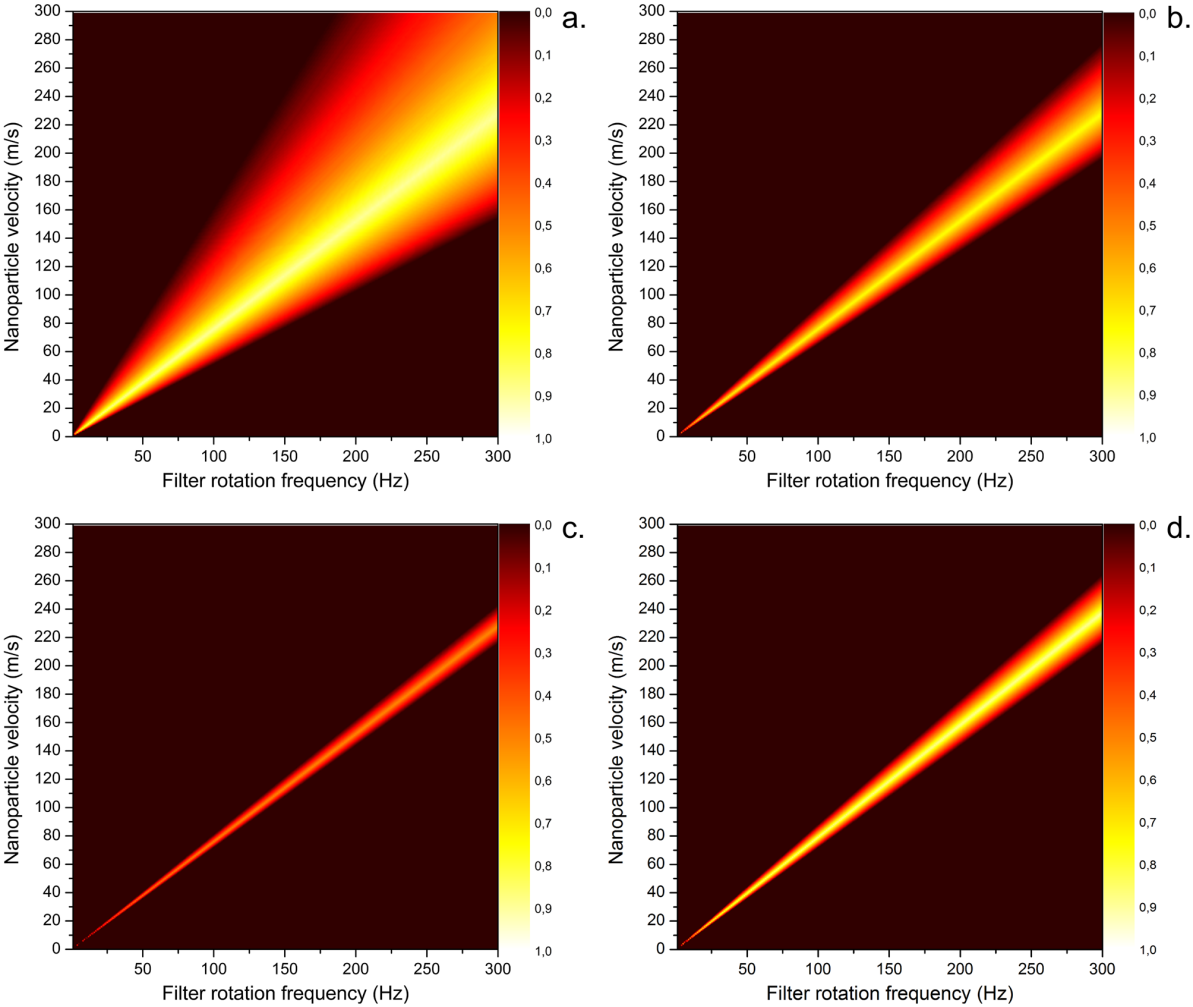
Calculated transmission signal maps for input nanoparticle velocities in the form of delta-functions, relative angle of the disks 9°, the distance of the disks 19 mm, disk thickness 1 mm and angular width of the slit (**a**) 5°; (**b**) 2°; (**c**) 1°. The last map under letter (**d**) shows the transmission of a helical rotor with a relative angle of the slit between the inlet and outlet of the slit again 9°, slit angular width 1° and thickness of the rotor 20 mm. The print resolution for the helical rotor (thickness of each subsequent incremental disk) is 0.2 mm.

At first, a profile of signal obtained with different angular widths of the slits has been investigated by simulation. If a δ-function in the velocity of the incoming particles is assumed, the profile of the signal is a triangle, the width of which is determined by the slit angular width and the velocity of the nanoparticles. This is shown in Fig. 4a–c, which presents the transmission functions obtained for the slotted disks systems with different angular widths of the slits *α*. In these figures, the horizontal cut of the map corresponds to one virtual measurement of the dependence of the number of detected particles on the rotational frequency of the filter with the velocity of NPs specified by the vertical position of the cut. On the other hand, the vertical cut shows the transmissivity of NPs velocities at the specific rotation frequency. Comparing the numerical results presented in Fig. 4a–c, it is clear that the lower values of *α* provide a narrower window of velocities at which nanoparticles at the given rotation frequency may pass the filter, i.e. better velocity resolution. For instance, while the range of nanoparticle velocities that contribute to the overall detected signal for the rotational frequency of 100 Hz and *α* equal to 5° is from 51 to 161 m/s, this range drops down to 72 to 81 m/s for the same rotational frequency and angular width of the slit 1°. However, the higher velocity resolution of the slotted disks system for the lower values of *α* is counterbalanced by the substantial decrease of the number of the NPs that pass the velocity filter, hence, to the lower signal. To give some example, the signal intensity, i.e. the integral of the number of NPs that went through the time-of-flight filter operated at the frequency of 100 Hz, decreased by a factor of 18 as the angular width of the slits changed from 5° down to 1°. The transmissivity of the 5° disks is 9.5%. The possible way how to reduce this unwanted effect is to increase the number of slits. Increasing the number of slits in case of the slotted disks rotor is, however, possible only to a certain degree. For the higher number of the slits, the nanoparticles (in the available frequency range) would start to pass through more than one slit in the second disk that gives rise to parasitic signal and thus complicates the analysis of the measured data. Therefore, if the input signal is too weak and the number of slits cannot be further increased without complications, the construction of the filter may be altered to helical channels, as suggested in^61,62^. The calculation for such a system shown in Fig. 4d matches a helical channel rotor printed with z-resolution 0.2 mm or, as mentioned earlier, a set of disks with thickness 0.2 mm set on top of each other with each rotated by an incremental angle. Such a helical rotor allows placing the slits/channels very close to each other since there may be no switching of the channel once the nanoparticle enters. With respect to the mechanical toughness of the whole rotating system, the ratio of particles that enter the helical rotor with 1° slits may be increased up to about 25% or even higher for wider slits.

At this point, it is worth noting that although the correction for finite disk thickness coming from Eq. (12) may seem insignificant, it cannot be neglected even for wider slits such as 5° (and disk thickness 1 mm) and becomes very significant for narrow slits. Neglecting of the correction causes an improper widening of the signal peak.

Figure 4c,d both show the calculation for slit width 1°, but in the first case, the disk thickness is 1 mm, while in the other only 0.2 mm. The output signal for the thinner disks is considerably more intense and with a broader peak.

**Figure 5:**
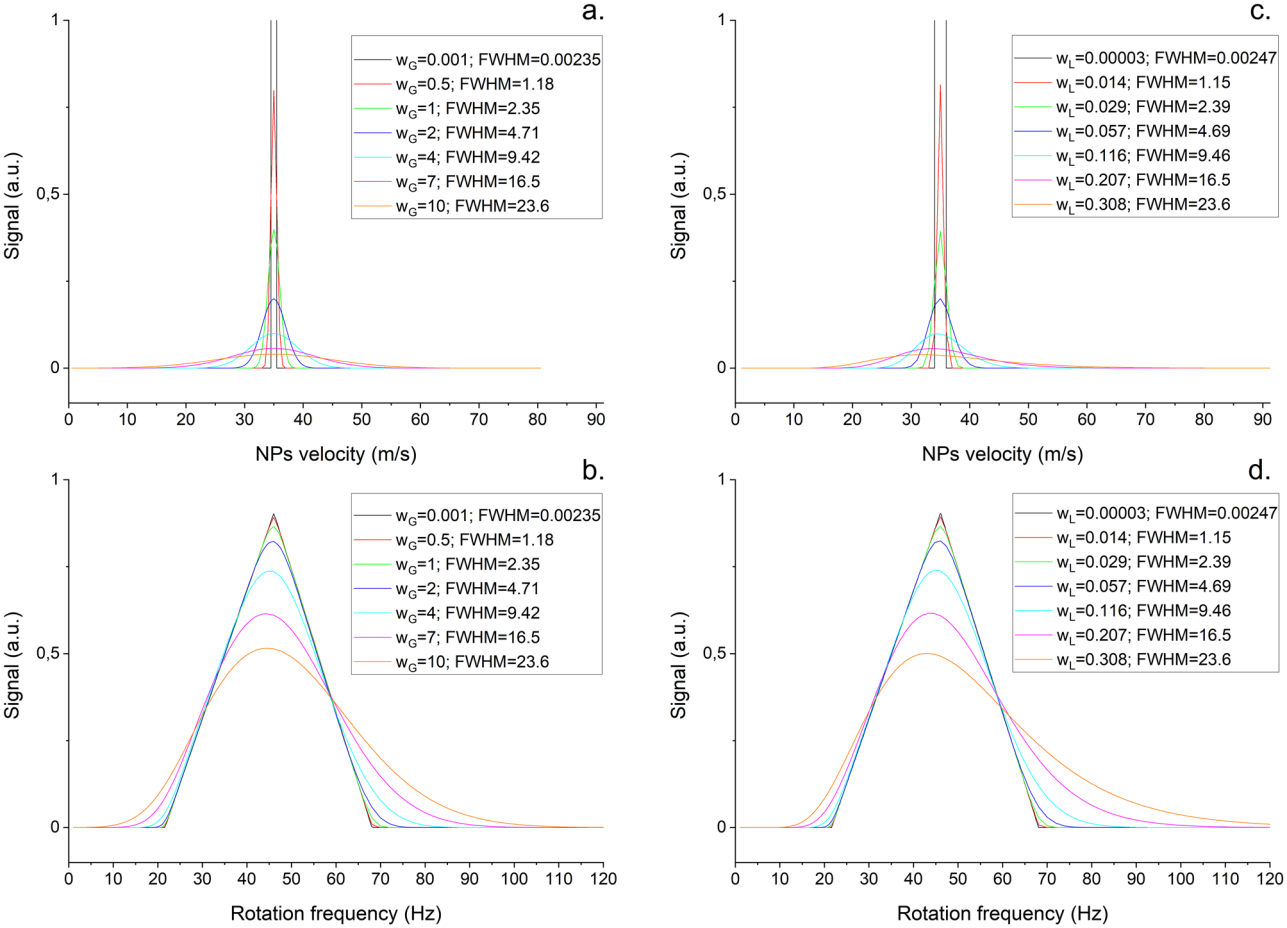
(**a**) Gaussian velocity distributions; (**b**) Simulation of the signals obtained from 5° disks for Gaussian velocity distributions; (**c**) log-normal velocity distributions. (**d**) simulation of the signals obtained from 5° disks for log-normal velocity distributions.

The aforementioned calculations supposed δ-function of the velocity of the incoming nanoparticles. However, this is non-realistic as certain velocity distribution has to be assumed. This leads to the widening of the signal peak width and the triangle edges are getting smoothed. To demonstrate this, examples of signals simulated using Eq. (14) for Gaussian (normal) and log-normal velocity distributions with fixed peak velocity *v*_*peak*_ and different full width at half maximum (FWHM) are presented in Fig. 5. In these calculations, the Gaussian distribution of the NPs velocities *v* is assumed in the form:

16 $${f}_{Gauss}\left(v\right)=\frac{{A}_{G}}{{w}_{G}\sqrt{2\pi }}{\exp}\left(-\frac{{\left(v-{v}_{peak}\right)}^{2}}{2{w}_{G}^{2}}\right)$$

where *A*_*G*_ is a scale factor connected to the peak height of the distribution and *w*_*G*_ is a so-called scale parameter, which is connected to the width of the distribution, while its FWHM is defined as:

17 $$FWHM=2{w}_{G}\sqrt{2{\ln}2}\approx 2.3548{w}_{G}$$

Analogously, the log-normal distribution is assumed in the form:

18 $${f}_{Lognormal}\left(v\right)=\frac{{A}_{L}}{{w}_{L}v\sqrt{2\pi }}{\text{exp}}\left(-\frac{{\left({\ln}{v}_{peak}-{\ln}v\right)}^{2}}{2{w}_{L}^{2}}\right)$$

where *A*_*L*_ is a scale factor connected to the peak height of the distribution and *w*_*L*_ is a real number parameter related to the width of the distribution. The FWHM of the distribution (18) may be derived in the form:

19 $$FWHM={\exp}\left(\left({v}_{peak}-{w}_{L}^{2}\right)+\sqrt{2{w}_{L}^{2}{\ln}2}\right)-{\exp}\left(\left({v}_{peak}-{w}_{L}^{2}\right)-\sqrt{2{w}_{L}^{2}{\ln}2}\right)$$

### Experimentally obtained velocity distribution functions

The detailed description of a simple numerical model of constructed time-of-flight velocity filters was presented in the previous section, with emphasis given to the description of the performance of such systems. This section will be devoted to the discussion of the experimental results and their analysis.

**Figure 6:**
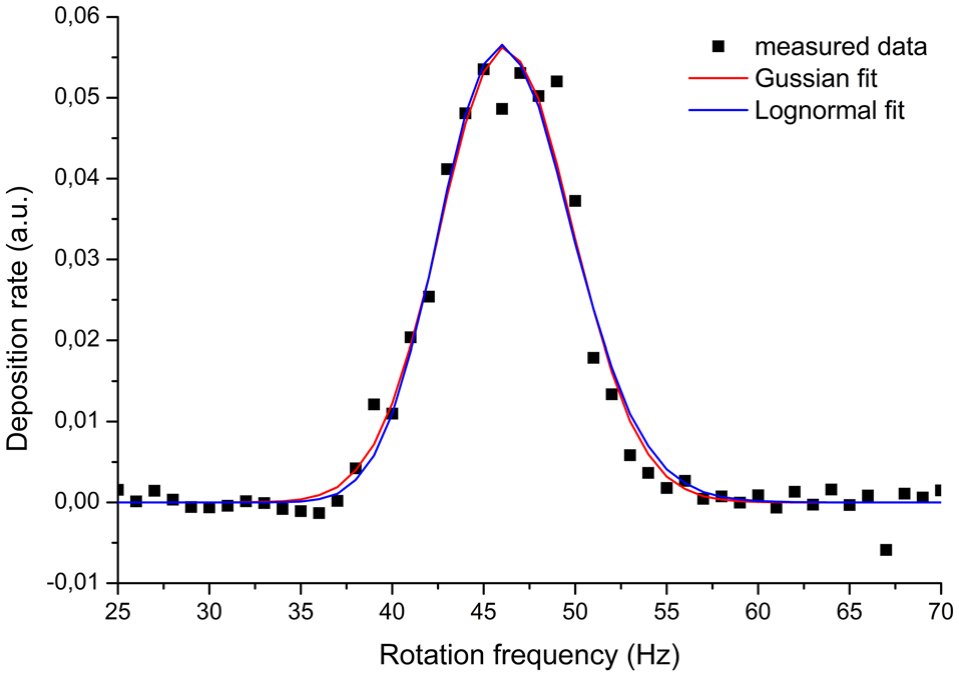
Comparison of fits using Gaussian (v_peak_ = 35.05 m/s; w_G_ = 2.624; FWHM = 6.18 m/s; RSS = 0.002813) and lognormal (v_peak_ = 35.08 m/s; w_L_ = 0.074; FWHM = 6.09 m/s; RSS = 0.002871) distributions of nanoparticle velocities. Nanoparticles have been deposited at aggregation chamber pressure 20 Pa, deposition chamber pressure 0.015 Pa, DC magnetron current 200 mA and QCM averaging over 20 values.

A typical example of a signal recorded by the QCM crystal, which is directly proportional to the number/mass of deposited NPs, in dependence on the rotation frequency is depicted in Fig. 6 for the disks with 1° slits and nanoparticles deposited at a pressure of 20 Pa in the aggregation chamber. It can be seen that under these experimental conditions, the measured data form a broad peak with a maximum value at the frequency of 46 Hz. Using the Eq. (15), this frequency value corresponds for a given geometry of the used time-of-flight filter to the nanoparticle velocity 35 m/s that may be considered as the mean velocity of nanoparticles in the main deposition chamber. This value is comparable with the previously reported velocities for similar systems and the equivalent size of NPs^49,63^. Furthermore, the width of the measured peak is considerably wider as compared to the peak simulated when the velocity of nanoparticles is assumed to be a δ-function. This suggests a velocity distribution with a finite width. Thus, to get a better insight into the real velocity distribution function, the experimental data have to be fitted using Eq. (14). Such fitting was performed using the calculated transmission function. The velocity distribution was supposed to be either Gaussian (16) or log-normal (18) with *v*_*peak*_, *A*_*G*_, *w*_*G*_ or *v*_*peak*_, *A*_*L*_, *w*_*L*_ left as free fitting parameters. The quality of the fit was evaluated by the residual sum of squares (RSS) and the distribution with the lowest RSS was taken as the result of the fit. The obtained results are presented in Fig. 6. As can be seen, both Gaussian and log-normal velocity distributions of nanoparticles may be used to fit the experimental data. Besides, both considered velocity distribution functions gave similar results showing the FWHM of the real velocity distribution to be 6.18 or 6.09 m/s, respectively. This example shows how the acquired experimental data, obtained according to the numerical model presented in the previous section may be converted into the velocity distribution function.

**Figure 7:**
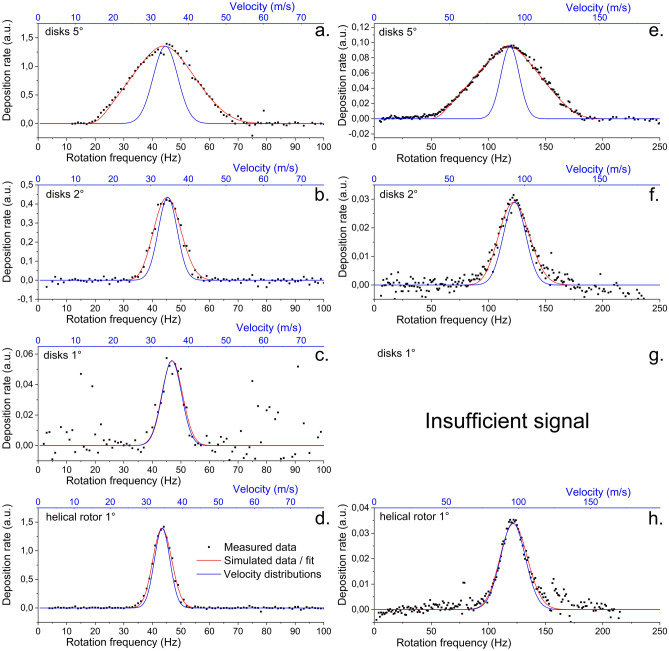
Comparison of the model with real measured data. (**a**–**d**) nanoparticles deposited at aggregation chamber pressure 20 Pa, deposition chamber pressure 0.015 Pa, DC magnetron current 200 mA and QCM averaging over 5 values; (**e**–**h**) nanoparticles deposited at aggregation chamber pressure 100 Pa, deposition chamber pressure 0.15 Pa, DC magnetron current 200 mA and QCM averaging over 20 values. The relative angle of the inlet and exit slit was 9°, disk distance 19 mm, disk thickness 1 mm, thickness of helical rotor was 20 mm printed with layer thickness 0.2 mm and the angular width of the slits was (**a**,**e**) disks 5°; (**b**,**f**) disks 2°; (**c**,**g**) disks 1° and (**d**,**h**) helical rotor 1°. The lower black axis is the directly measured frequency of the rotor, the upper blue axis is formally converted to velocity using Eq. (15). The fits of the measured data in red are accompanied by the derived NPs velocity distributions in blue.

Further tests were performed with all four rotors described above for two different deposition conditions (20 and 100 Pa pressure in the aggregation chamber) to investigate to what degree it is possible to reconstruct the NPs velocity distribution at different experimental conditions. The results of the measurements and their fits are shown in Fig. 7 (As the results obtained using both Gaussian and log-normal distribution gave always similar results, only the Gaussian distribution was considered.). Based on these data, the following conclusions may be drawn.

**Figure 8:**
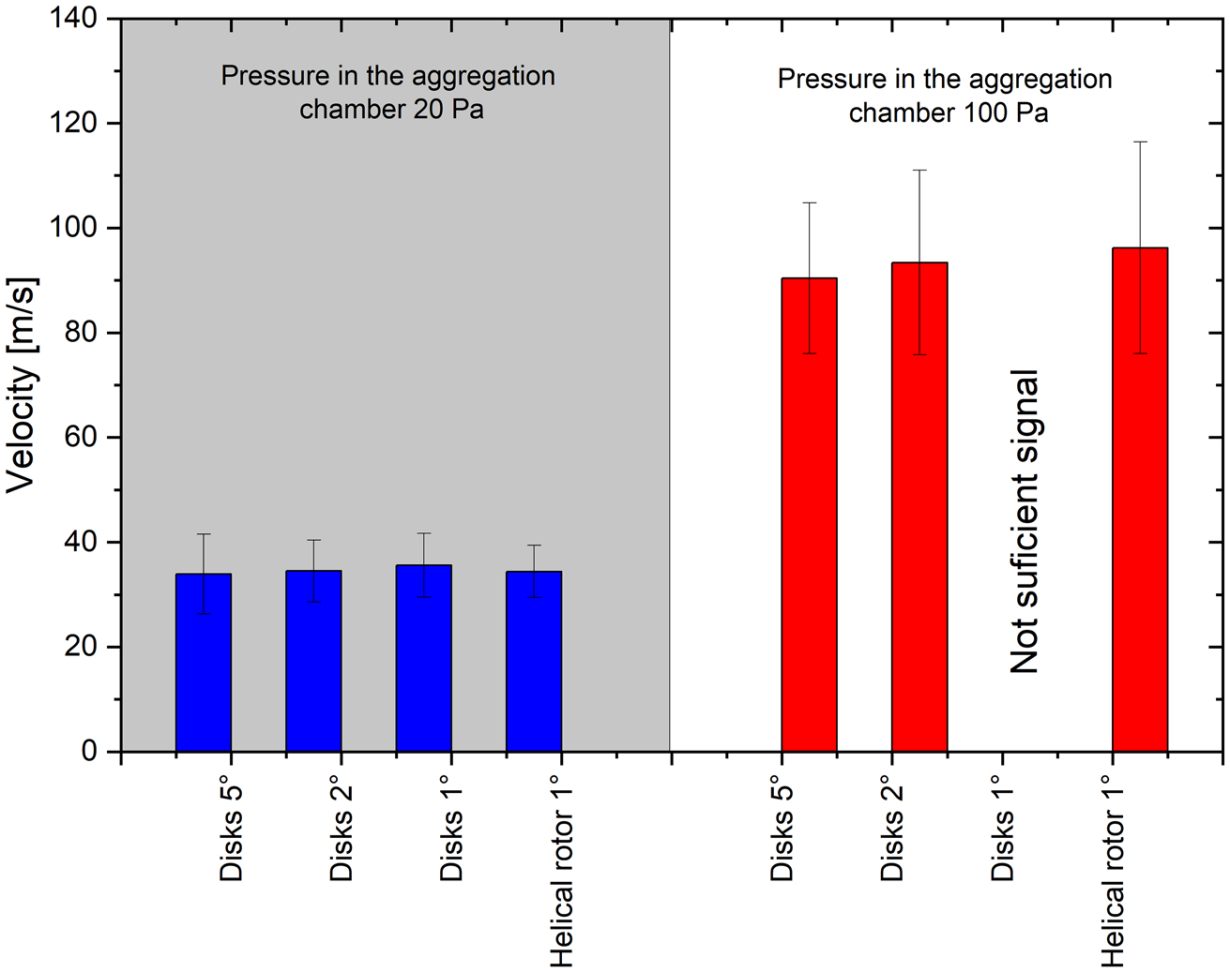
Fit parameters for measurements shown in Fig. 7. The bars show the mean velocity, while the error bars show the FWHM.

First, it is evident that whereas the position of the velocity peak in measured data is independent of the used rotor, it differs significantly for different pressures in the aggregation chamber of the mGAS system: in the case of pressure 20 Pa the mean velocity of NPs was found to be around 35 m/s, while for the pressure of 100 Pa the mean velocity was 93 m/s. Alongside with the constant values of mean velocities for fixed pressure in the aggregation chamber, also the FWHM of the velocity distribution function determined by the fitting of the experimental data stayed almost unchanged (see Fig. 8). The close values of the mean velocity of nanoparticles and FWHM of their velocity distribution for constant pressure in the aggregation chamber obtained by the different rotors of the time-of-flight filter demonstrate the good reproducibility of the measurement and robustness of the way, how the data were treated. It also demonstrates that the rotors themselves do not in any measurable way influence the velocity of the nanoparticle beam, otherwise there would have to be difference between disks and helical rotor. The small variations may be attributed to many factors such as rotors manufacturing accuracy and accuracy of mounting, or inevitable slight changes of conditions inside the nanoparticle source (e.g. variations of the gas temperature or on-going increase of the depth of the erosion track^64^) connected with the long-term operation of mGAS. On the other hand, the shift in the mean velocity of NPs towards higher values for higher pressure in the aggregation chamber is due to the higher velocity and density of the buffer gas that results in more intensive acceleration of the carried NPs. This is an important finding as it experimentally proves that the velocity of the nanoparticles that leave the mGAS may be easily controlled by the pressure/flow of the carrier gas.

The second important finding that can be demonstrated in Fig. 7 is the gradual decrease of the measured signal with the decreasing angular width of the system based on the slotted disks. This observation fully agrees with the numerical model. Furthermore, the decrease in the signal intensity is more important for the higher pressure at which it was not possible to get a measurable signal for 1° slits. This is due to the less efficient production of nanoparticles at higher pressures. In fact, the unobstructed deposition rates, i.e. the deposition measured by the QCM without the time-of-flight filter, are 15 Hz/s for the pressure of 20 Pa in the aggregation chamber, while the deposition rate of 1 Hz/s was observed when the pressure was increased up to 100 Pa. However, even at such a low deposition rate that corresponds to the one monolayer of NPs in the center of the deposition spot in approximately 15 min, a measurable signal was obtained for all rotors except the disks with 1° slits. This proves the capability of such a system to measure velocities even at low nanoparticle fluxes.

Finally, gradual narrowing of the measured velocity peak with lowering the angular width *α* of the slits was observed and the measured data approached for the lower values the real velocity distribution function. This effect, which is in agreement with the numerical model of the time-of-flight filter performance, has an important practical consequence as it clearly shows that in the case of low values of *α* the velocity distribution function with a sufficient level of precision may be obtained directly from the measured data using Eq. (15), i.e. without the necessity of the laborious fitting of the experiment data.

## Conclusions

The time-of-flight filters based either on slotted disks or on a helical rotor were found to be despite their relative simplicity a powerful tool for the precise measurement of velocities and velocity distributions of nanoparticles leaving the mGAS system. As shown in this study, such devices may be used in several different configurations maximizing simplicity, signal amplitude or resolution. Furthermore, it is demonstrated that irrespective of the particular configuration of the measuring setup and with it connected velocity resolution, the recorded experimental data may be easily recalculated to the real velocity distribution functions. This allows one to select a proper design for a particular application, including the possibility to perform the velocity measurements even at the conditions that provide deposition rate as low as 1 monolayer of NPs per several minutes. This is an important finding as it paves the way for the better characterization of beams of nanoparticles generated by gas aggregation sources, which is urgently needed in various applications in which the velocity/energy of nanoparticles plays an important role. In addition, such measured velocity distribution functions may be used as a benchmark for testing of the validity of the theoretical models.

## Data Availability

All data and models generated or used during the study are available on request.
